# Modelling phagosomal lipid networks that regulate actin assembly

**DOI:** 10.1186/1752-0509-2-107

**Published:** 2008-12-05

**Authors:** Mark Kühnel, Luis S Mayorga, Thomas Dandekar, Juilee Thakar, Roland Schwarz, Elsa Anes, Gareth Griffiths, Jens Reich

**Affiliations:** 1 EMBL, Postfach 102209, 69117 Heidelberg, Germany; 2 Laboratorio de Biología Celular y Molecular, IHEM-CONICET, Facultad de Ciencias Médicas, Universidad Nacional de Cuyo, Mendoza, Argentina; 3 Lehrstuhl für Bioinformatik, Biozentrum, Am Hubland, D-97074 Würzburg, Germany; 4 Department of Physics, 104 Davey Laboratory, Pennsylvania State University, University Park, PA 16802, USA; 5 Institut für Hygiene und Mikrobiologie, Josef-Schneider-Straße 2/Bau E1 97080 Würzburg, Germany; 6 Molecular Pathogenesis Centre, Unit of Retrovirus and Associated Infections, Faculty of Pharmacy, University of Lisbon, Av. Forcas Armadas, 1600-083 Lisbon, Portugal; 7 Max Delbrück Center, PO Box 74023810, D-13092 Berlin, Germany

## Abstract

**Background:**

When purified phagosomes are incubated in the presence of actin under appropriate conditions, microfilaments start growing from the membrane in a process that is affected by ATP and the lipid composition of the membrane. Isolated phagosomes are metabolically active organelles that contain enzymes and metabolites necessary for lipid interconversion. Hence, addition of ATP, lipids, and actin to the system alter the steady-state composition of the phagosomal membrane at the same time that the actin nucleation is initiated. Our aim was to model all these processes in parallel.

**Results:**

We compiled detailed experimental data on the effects of different lipids and ATP on actin nucleation and we investigated experimentally lipid interconversion and ATP metabolism in phagosomes by using suitable radioactive compounds.

In a first step, a complex lipid network interconnected by chemical reactions catalyzed by known enzymes was modelled in COPASI (Complex Pathway Simulator). However, several lines of experimental evidence indicated that only the phosphatidylinositol branch of the network was active, an observation that dramatically reduced the number of parameters in the model. The results also indicated that a lipid network-independent ATP-consuming activity should be included in the model. When this activity was introduced, the set of differential equations satisfactorily reproduced the experimental data. On the other hand, a molecular mechanism connecting membrane lipids, ATP, and the actin nucleation process is still missing. We therefore adopted a phenomenological (black-box) approach to represent the empirical observations. We proposed that lipids and ATP influence the dynamic interconversion between active and inactive actin nucleation sites. With this simple model, all the experimental data were satisfactorily fitted with a single positive parameter per lipid and ATP.

**Conclusion:**

By establishing an active 'dialogue' between an initial complex model and experimental observations, we could narrow the set of differential equations and parameters required to characterize the time-dependent changes of metabolites influencing actin nucleation on phagosomes. For this, the global model was dissected into three sub-models: ATP consumption, lipid interconversion, and nucleation of actin on phagosomal membranes. This scheme allowed us to describe this complex system with a relatively small set of differential equations and kinetic parameters that satisfactorily reproduced the experimental data.

## Background

When trying to understand complex signalling networks, success depends on the ability of the investigator to access the system in order to manipulate components and test hypotheses; for this, a simple experimental system is needed. For several years we have used latex bead phagosomes (LBP) for *in vitro *and *in vivo *analyses of complex membrane processes, due to the ease by which these organelles can be isolated in pure form from macrophages [1]. An additional advantage of these organelles for analyzing signalling networks is that the cytosolic side of the membrane is exposed to the investigator. LBP represent the simplest imaginable membrane system, a single bilayer around a 1 μm latex bead. Nevertheless these organelles are of amazing complexity, being composed of at least 1000 so far identified proteins (Desjardins, personal communication) as well as hundreds, if not thousands of lipids (Brouwers et al, personal communication). Among these are functional units of proteins and lipids that sense incoming signals, regulate fusion, and interact with the cytoskeleton [1]. Since all these processes have been reconstituted *in vitro *[2-5], the intricate membrane machinery required for their operation must be functionally intact on these isolated membrane organelle.

In this study, we addressed the modulation by ATP and lipids of the process by which LBP nucleate actin *in vitro*. In this process, actin filaments grow out perpendicularly from the membrane. On phagosomes, as in all known examples of membrane-catalyzed actin assembly, actin monomers are inserted at the membrane surface [6]. This is a cytosol- and GTP-free reaction, requiring only G actin, thymosin β4 (which works as a monomeric actin buffer), and ATP. A number of proteins have been implicated in this process, most prominently ezrin and/or moesin [7].

Interestingly, the process is highly sensitive to the lipid composition of the membrane: addition of specific lipids leads to pronounced differences in the response of phagosomes to actin assembly, in a manner that varies with ATP levels [4,8]. Phagosomes are metabolically competent, as they contain enzymes and metabolites necessary for lipid interconversion [4,8-11]. Therefore, the addition of ATP and lipids to the incubation will provoke a perturbation of the steady-state phagosomal membrane composition attained after preparation of the organelle in a metabolite-free medium. The dynamic rearrangement of the metabolic state occurs simultaneously with the nucleation of actin and the filament growth. Our aim here was to model both processes, metabolic changes of ATP and lipids as well as actin polymerization, in parallel. Towards this goal, we compiled detailed experimental data on the effects of different lipids and ATP on phagosome actin assembly, and investigated lipid interconversion and ATP metabolism in phagosomes experimentally by using suitable radioactive compounds. All the results were integrated into a mathematical model that combined three independent sub-models: ATP consumption, lipid interconversion, and nucleation of actin on phagosomal membranes. This division allowed to describe this complex system with a relatively small set of differential equations and kinetic parameters which satisfactorily reproduced the experimental data.

## Results

### Fate of ATP during incubation with phagosomes

For this study, we used purified phagosomes made by internalizing 1 μm latex beads, to which avidin had been covalently attached, for 1 h pulse followed by 1 h chase. The addition of 1 mM ATP to 2 h LBP induces the phosphorylation of many (unidentified) proteins by phagosomal kinases [11]. ATP concentration also strongly affects actin polymerization by phagosomes [7]. In addition, this nucleotide is a 'hub' molecule in several metabolic routes, including phospholipid metabolism. We therefore studied the fate of ATP in our phagosomal preparation. Upon addition of γ^32^P-labelled ATP (γ32P.ATP), radioactive phosphate was incorporated into several phospholipids (see below), and presumably into proteins, nucleotides and other metabolites. However, this accounted only for a negligible fraction (less than one percent) of the added ATP. The bulk of ^32^P appears as orthophosphate and may be the consequence of unspecified ATPase-like reactions in the phagosome membrane.

**Figure 1:**
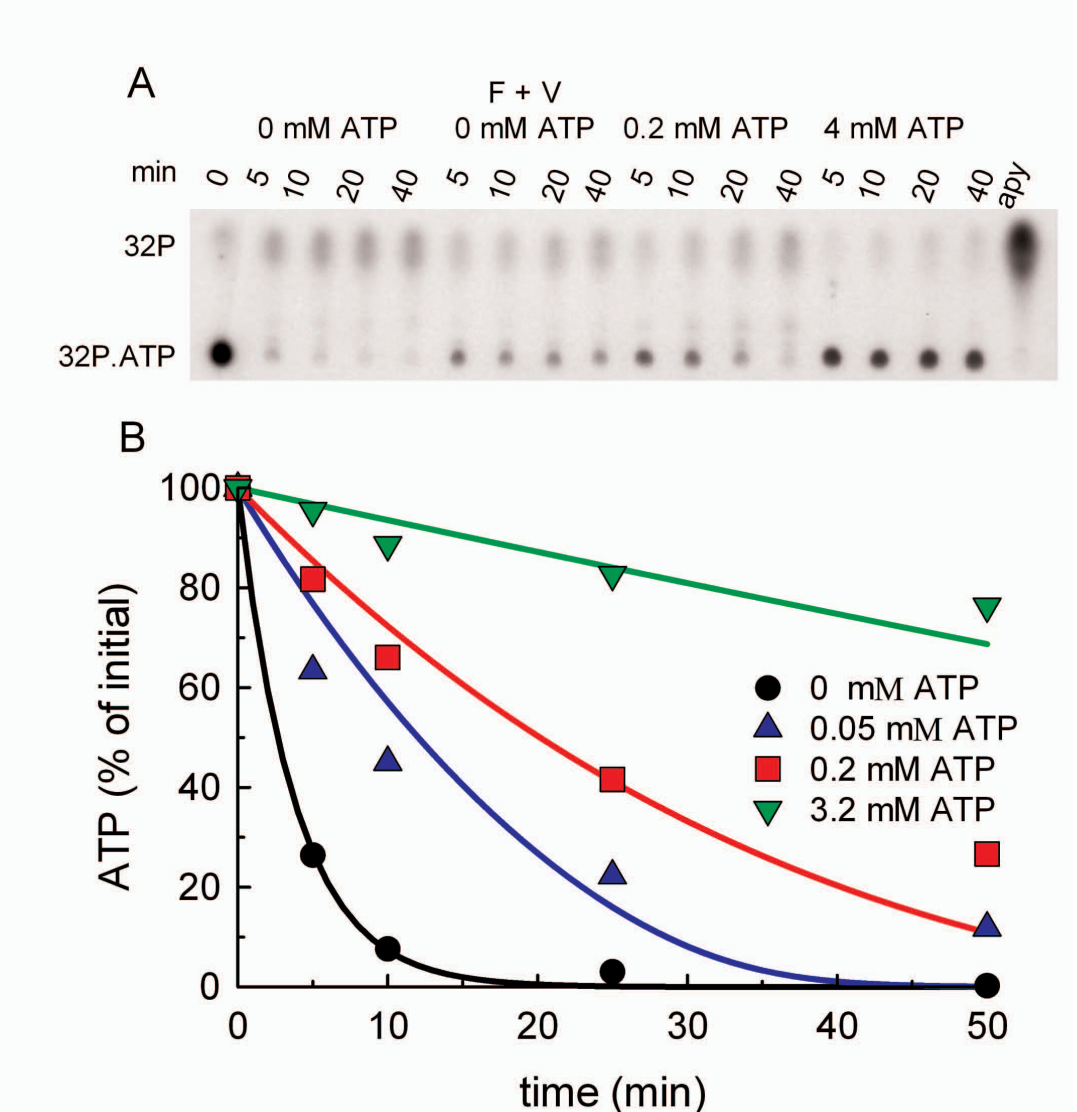
**ATP consumption by phagosomal preparations**. Phagosomal preparations were incubated al 20°C with γ32P.ATP in the presence of different concentrations of unlabelled ATP and with the addition (when indicated) of 10 mM KF + 1 mM orthovanadate (F + V). Lipids and proteins were separated by a chloroform:methanol extraction and the water soluble radioactive species were resolved by TLC. A. A typical TLC plate showing the time dependent ATP hydrolysis. Left most lane: γ32P.ATP (5X concentration) before adding to the phagosomal preparation. Right most lane: same γ32P.ATP aliquot incubated with 10 U/ml apyrase. B. The TLC plate of a typical experiment as shown in A was scanned and the percentage of non hydrolyzed γ32P.ATP was estimated. Experimental data are plotted as symbols; the model predictions as solid lines having the same colours than the corresponding experimental observations.

Phagosomes were incubated under the same conditions used for actin polymerization and a fixed amount of γ32P.ATP was added in the presence of varying concentrations of unlabelled ATP. Lipids and proteins were discarded following a methanol:chloroform extraction, and free radiolabelled orthophosphate was separated from ATP by TLC. In Fig. 1A, a typical experiment is shown. Two non-specific phosphatase inhibitors, fluoride and vanadate, inhibited γ32P.ATP hydrolysis, as well as addition of unlabelled ATP (Fig. 1A). Apyrase completely degraded ATP within 5 min. The time course of the γ32P.ATP hydrolysis, expressed as a percentage of the respective initial value at different concentrations of unlabelled ATP is shown in Fig. 1B. The decay rate apparently followed a two-component exponential function. The rate of ATP degradation showed a non-linear dependence on the initial concentration of unlabelled ATP, suggesting saturable ATP-consuming reactions. We decided to fit the rates to Michaelis-Menten-type kinetics. The decay curves were fitted to the sum of two reactions with different characteristics; one reaction that occurs already at low ATP concentrations and has a low maximal velocity, and a second reaction which becomes important only at much higher ATP concentration but has a higher maximal velocity. The corresponding differential equation has the following form:

dATP/dt = - v_h _- v_k_

with

v_h _= V_h _* ATP/(ATP + K_h_) and v_k _= V_k _* ATP/(ATP+ K_k_)

where

v_h _and v_k _(ATP decay rates)

ATP (actual ATP concentration at time t)

V_h _and K_h _(maximal rate and concentration for half-maximal rate of the rapid ATP decay)

V_k _and K_k _(maximal rate and concentration for half-maximal rate of the slow ATP decay)

The differential equation was solved for specified parameter values and initial concentrations by using the Complex Pathway Simulator (COPASI) software (see Additional file 1). A parameter estimation routine, also performed by COPASI, allowed a search for V_h_, K_h_, V_k_, and K_k _values to produce an optimal fit (defined as minimal sum over all squared differences between experimental and predicted concentrations). The results are indicated in Table 1 together with an estimator of the sensitivity of the fitting to parameter perturbation (described in Methods).

**Table 1:** Parameter estimation for ATP consumption (sub-model I)

Parameter	units	Value	sensitivity (ΔlnSS/Δlnp)
V_h_	μmol/(l*min)	24.3	0.272
K_h_	μmol/l	758	0.046
V_k_	μmol/(l*min)	1.08	0.069
K_k_	μmol/l	4.72	0.072

The results of two independent γ32P. ATP hydrolysis experiments and the COPASI program were used to estimate parameters. Sensitivity for each parameter was calculated as explained in Methods.

As shown in Fig. 1B, the coincidence of prediction and measurement was satisfactory, with a tolerable systematic scatter of experimental values around the model prediction (for clarity, only one of the two experiments used for the calculations is plotted). The deviation of theory from the experimental data was about 14%, using squared deviations, or 10%, using absolute deviations as criterion (see Methods). In conclusion, the ATP concentration in the system showed dynamic changes, implying that actin polymerization occurred in an 'evolving' environment. This time-dependent ATP consumption was taking into account when the lipid network and the assembly of actin filaments were modelled (see below).

### Lipid network dynamics

**Figure 2:**
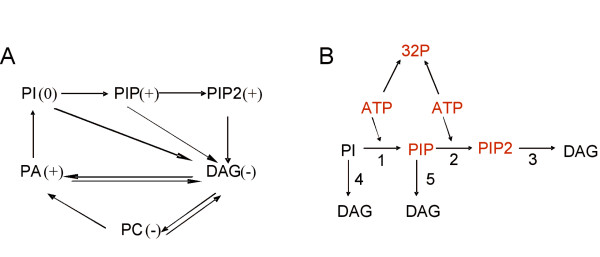
**A simplified lipid network interconnecting several species that affect actin nucleation on purified phagosomes**. The qualitative effect on actin nucleation is shown in brackets (+, activation; -, inhibition; 0, no effect). B. Branch of the network shown in A that was found to be active in purified phagosomal fractions. The numbers refer to the reactions in sub-model II. In red are the radioactive species detected in the experimental assays (see Fig. 1 and 3).

**Figure 3:**
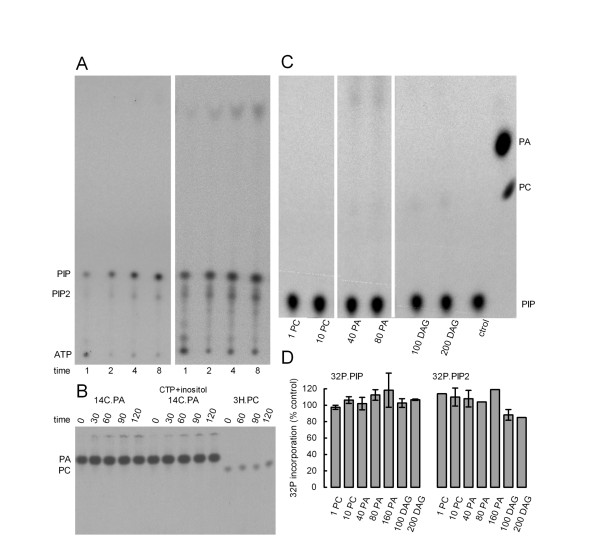
**Phospholipid metabolism in phagosomal preparations**. A. A phagosomal preparation was incubated with γ32P.ATP and 0.1 mM unlabelled ATP at 20°C for different periods of time (min). Phospholipids were extracted with chloroform:methanol and resolved by TLC. The plate was exposed for 8 h (left) or 24 h (right). B. A phagosomal preparation was incubated with ^14^C(glycerol).PA (14C.PA) or ^3^H(choline).PC (3H.PC), 0.2 mM unlabelled ATP and (when indicated) 1 mM CTP and 1 mM inositol for different periods of time (min). Phospholipids were extracted and resolved by TLC. C. A phagosomal preparation was incubated with γ32P.ATP and 0.2 mM unlabelled ATP in the presence of different concentrations of unlabelled PC, PA, and DAG (μM) for 15 min. Phospholipids were extracted and resolved by TLC. Radiolabelled PA and PC were included in the right most lane as markers. Only the upper part of the TLC plate is shown. D. In experiments similar to the one shown in C, different concentrations of unlabelled lipids (μM) were included in the assay. The amount of ^32^P incorporated in PIP and PIP2 was quantified and expressed as a percentage of the incorporation observed when no lipid was added. The values represent the mean of 2 or 3 independent experiments (error bars: range for N = 2, SEM for N = 3).

From previous experiments we knew that certain phosphorylated and non-phosphorylated lipids modulate actin assembly [8,9]. Fig. 2A shows a simplified metabolic network based on accepted biochemical knowledge  that connects several lipids affecting actin polymerization on phagosomal membranes. In this scheme, except for diacylglycerol (DAG), all other molecules are phospholipids. We also summarize the effect on actin filament assembly of these lipids when added to the system (symbols in brackets). It is impossible to decide *a priori *whether any of the effects on actin assembly is direct or indirect (i.e., through conversion of one lipid into another directly-acting metabolite during the incubation in the presence of ATP). Therefore, to separate the effect of lipids, it was necessary to first assess the dynamics of these molecules in the isolated phagosomes. A powerful method to analyze the phospholipid metabolism in a system is to follow the incorporation of ^32^P from the γ-phosphate of radiolabelled ATP. We therefore performed experiments where γ32P.ATP was incubated during different periods of time with phagosomal preparations under the same conditions as used for actin polymerization. Lipids were extracted with chloroform:methanol and radioactive lipids resolved by TLC. Three prominent spots were consistently seen, corresponding to the remaining ATP (staying at the origin), and to phosphatidylinositol-4-phosphate (PIP) and phosphatidylinositol-4,5-bisphosphate (PIP2, Fig. 3A). The optical density of these spots is approximately proportional to the amount of the radiolabelled compounds at the end of the incubation. The concentration of PIP was always higher than that of PIP2, in agreement with previously published results [4]. Only few radioactive compounds were observed migrating faster than PIP, even after over-exposing the TLC plates (Fig. 3A, right set of lanes). The absence of bands in the locations of phosphatidic acid (PA), phosphatidylinositol (PI) and phosphatidylcholine (PC) -the other phosphate-containing lipids in the network, that should migrate above PIP in this TLC system- showed that there was little incorporation of ^32^P into these compounds; the kinases and transferases required for their synthesis are likely inactive. An unidentified band was seen in the solvent front and its identification was not pursued further. To confirm that the branches involving PA and PC are less active, we incubated ^14^C.PA or ^3^H.PC with phagosomes for extended periods of time. Fig. 3B shows that during incubation neither lipid was metabolized to other species indicating that the conversions of PA to PI or DAG, as well as of PC to PA or DAG were not active. PA did not change even if CTP and inositol (cofactors of the metabolic pathway towards PI) were added to the assay (Fig. 3B). We further tested whether the addition of unlabelled PA, PC or DAG could affect the incorporation of ^32^P into phosphorylated lipids. PC had no effect while PA and DAG slightly increased the incorporation of ^32^P into the fast moving lipid and into an unidentified phospholipid migrating close to PC on the TLC plate (Fig. 3C). However, up to 200 μM DAG did not promote any significant increase of radiolabelled PA, even after exposing the TLC plates for long periods of time (Fig. 3C). This observation indicates that DAG is not actively phosphorylated to PA in the phagosome by a DAG kinase activity. Fig. 3D shows that relatively high concentrations of PC, PA, or DAG, all of which affected actin polymerization (see below) only slightly altered the synthesis of PIP and PIP2.

We also tested whether addition of soluble cofactors that participate in lipid interconversion could promote ^32^P incorporation. To this end, CTP, choline, and inositol were added in combination with PA or DAG. However, labelling of PI or PC was not observed (data not shown). The only relevant effect was that CTP inhibited the incorporation of ^32^P into PIP and PIP2 (data not shown). This is likely to be due to a dilution effect, caused by ATP synthesis from unlabelled CTP by nucleoside diphosphokinase.

In conclusion, the observations with labelled and unlabelled lipids indicate that the only network branch metabolically active in the 2 h LBP is the synthesis and consumption of PIP and PIP2. The dynamically relevant phospholipid subsystem is depicted in Fig. 2B. The enzyme reactions indicated by numbers 1 through 5 -together with documented *in vitro *kinetic parameters [12-14]- are:

Reaction 1: PI-4kinase (2.7.1.67 Km = 115 μM for PI; Km = 150 μM for ATP)

Reaction 2: PIP-5kinase (2.7.1.68; Km = 5 μM for PIP; Km = 19 μM for ATP)

Reaction 3: phosphoinositide phospholipase C (3.1.4.11; Km = 170 μM for PIP2)

Reaction 4: phosphoinositide phospholipase C (3.1.4.11; Km = 130 μM for PI)

Reaction 5: phosphoinositide phospholipase C (3.1.4.11; Km = 310 μM for PIP)

We derived a system of differential equations describing the time course of those metabolites that change concentration during incubation. In this system, ATP consumption was described as an independent subsystem with reactions v_h _and v_k_, as parameterized above. The fitting of ATP's influence on kinase reactions 1 and 2 required saturable (Michaelis-Menten) kinetics. Simple linear rate laws did not produce satisfactory 'fits' (results not shown). Moreover, the published Km for reaction 2 was in the micromolar range for PIP; a value that is likely lower than the actual lipid concentration in the membranes when this compound is added to the system (see below). Hence, a saturable kinetics was used for PIP. For the other reactions, mass-action kinetics proved satisfactory. The resulting system of differential equations describing this system reads:

d(PI)/dt = - v_1 _- v_4 _(PI is being consumed from an initial concentration)

d(PIP)/dt = v_1 _- v_2 _- v_5 _(PIP is being produced by one and consumed by two reactions)

d(PIP2)/dt = v_2 _- v_3 _(PIP2 is being produced and consumed)

d(DAG)/dt = v_3 _+ v_4 _+ v_5 _(DAG accumulates during the incubation)

with

v_1 _= V_1 _* PI * ATP/(ATP+K_1_)

v_2 _= V_2 _* PIP/(PIP+K_3_) * ATP/(ATP+K_2_)

v_3 _= k_3 _* PIP2

v_4 _= k_4 _* PI

v_5 _= k_5 _* PIP

where the k's are rate parameters, the K's are Michaels half-maximal activity parameters, V's are maximal rates, and the metabolite acronyms (PI, PIP, PIP2, DAG, ATP) stand for their time-variable concentrations. Initial lipid concentrations in the phagosomal membrane were estimated as 266 μM PC, 49 μM PI, 2.5 μM PIP, 0.25 μM PIP2, 10 μM PA, 7.4 μM DAG (see "Phagosomal lipid assumptions" in Methods). The uncertainty in these values is considerable. Fortunately, it turned out that our conclusions were not strongly affected by changes in the initial lipid concentrations (see below).

**Figure 4:**
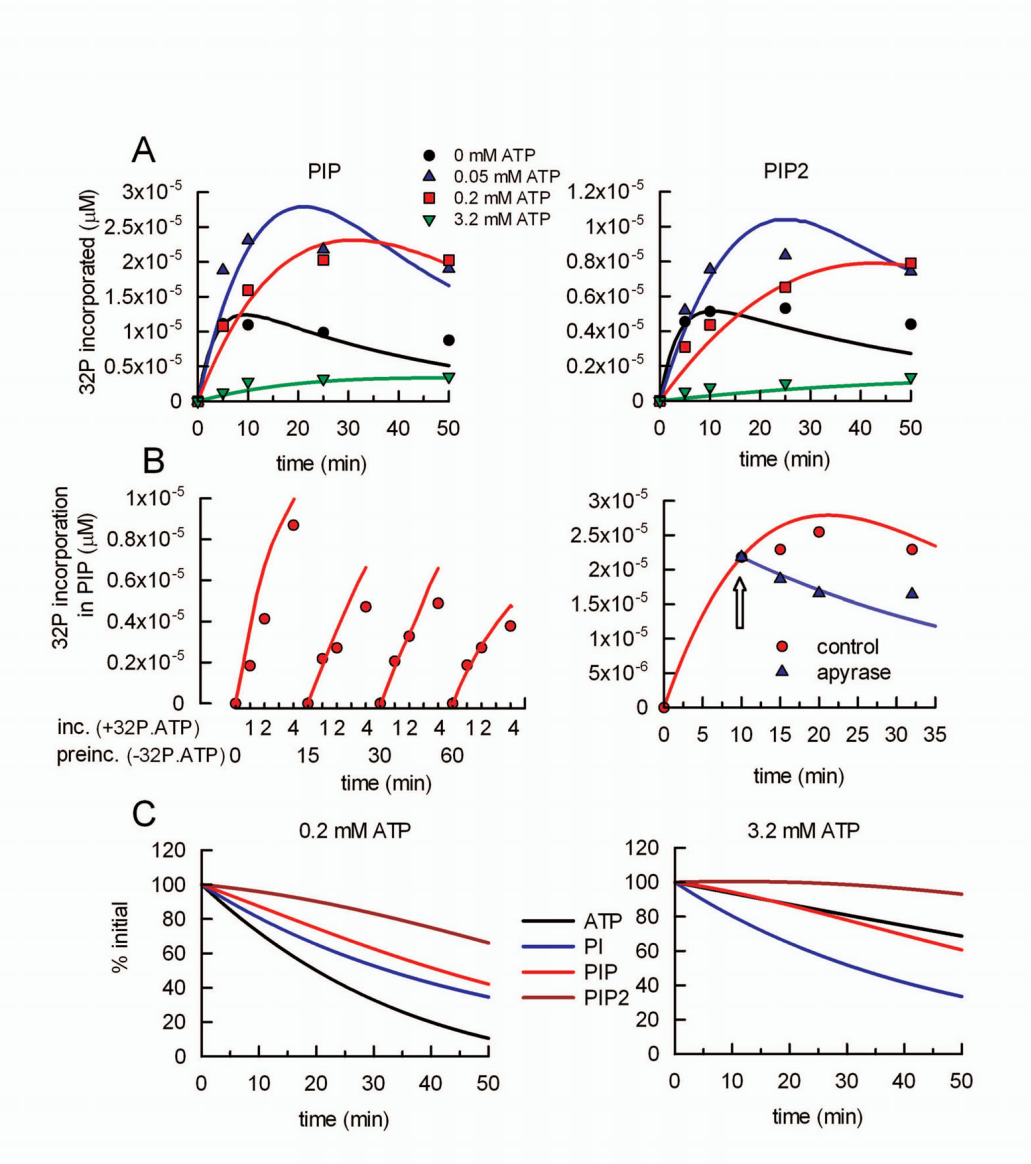
**Experimental and model values for ^32^P incorporation in PIP and PIP2**. A. Phagosomal preparations were incubated with γ32P.ATP at 20°C for different periods of time in the presence of different concentrations of unlabelled ATP. Phospholipids were extracted with chloroform:methanol and resolved by TLC. The radioactivity incorporated in PIP and PIP2 was measured and calibrated with a γ32P.ATP standard. B. Left panel. Phagosomal preparations were pre-incubated with 0.2 mM cold ATP at 20°C for 0, 15, 30 or 60 min. Afterwards, γ32P.ATP was added and the samples were incubated for 0, 1, 2, and 4 min. Right panel. Phagosomal preparations were incubated with γ32P.ATP in the presence of 50 μM unlabelled ATP for 10 min at 20°C. Apyrase (0 or 1 U/ml) was then added to samples (arrow) and the incubation continued for 0, 5, 10, or 22 min. Radiolabelled PIP was measured as in A. For A and B, experimental data are plotted as symbols; the model predictions as solid lines having the same colours than the corresponding experimental observations. C. Model predicted variation of ATP and phospholipids at two concentration of ATP: 0.2 mM (left panel) and 3.2 mM (right panel).

With rate parameters and initial conditions specified, the software COPASI solves the time-course for all implicated metabolites (see Additional file 2). Furthermore, a parameter estimator (in COPASI) can fit the parameters to describe the actually measured time-concentration matrix of PIP and PIP2 in the least-squares' sense. For this, the time courses of ^32^P incorporation into PIP and PIP2 at different ATP concentrations were used. The results of only two experiments were included for parameter estimation; however, qualitatively similar results were obtained in a total of five independent experiments. The results of parameter estimation are given in Table 2 together with an estimator of the sensitivity of fitting to parameter perturbations. Fig 4A shows fitted and measured ^32^P-labelled PIP and PIP2 values (for clarity, only one of the two experiments used for the calculations is plotted). The deviation of the model from the experimental data was estimated to be about 18% (squared deviations) or 15% (absolute deviations), which indicates a tolerable systematic scatter of experimental values around the model prediction.

**Table 2:** Parameter estimation for the lipid network (sub-model II)

Parameter	units	Value	sensitivity (ΔlnSS/Δlnp)
V_1_	1/min	0.00106	0.455
K_1ATP_	μmol/l	159	0.354
V_2_	μmol/(l*min)	0.0139	0.575
K_2ATP_	μmol/l	62.9	0.424
K_2PIP_	μmol/l	5.00	0.051
k_3_	1/min	0.0175	0.361
k_4_	1/min	0.0209	0.154
k_5_	1/min	0.0226	0.454

The results of two independent experiments of ^32^P-incorporation into lipids and the COPASI program were used to estimate parameters. Sensitivity for each parameter was calculated as explained in Methods.

Several other experiments were performed to test the model. In one experiment, phagosomes were incubated with only unlabelled ATP and after different times of pre-incubation, γ32P.ATP was added and incorporation of ^32^P into PIP was analyzed (Fig. 4B, left panel). In a different set of experiments, after 10 min incubation with γ32P.ATP, apyrase was added to the system and the labelling in PIP was followed for several minutes (Fig. 4B, right panel). The model agreed well with the experimental results, indicating that the reactions included in the model and the parameters selected are a good description of the phagosomal lipid network. Note that in the case of the enzyme kinetic K parameters, the estimates are in reasonably good agreement with the reported Km constants. Note also that the three phospholipase C (PLC)-like reactions (reactions 3, 4, and 5, Fig. 2B) have similar k parameters. Moreover, the sum of squared deviations between data and prediction was not significantly affected when a single k parameter was used for all three phosphatases.

The model predicts the following time course characteristics for radioactive lipid dynamics that accurately reproduce the experimental observations (Fig. 3): 1. ^32^P is continuously incorporated in PIP and PIP2, a process that is limited by the consumption of γ32P.ATP. 2. Most of the ATP consumption is caused by the ATPase reactions included in the model. 3. At low ATP concentrations, the nucleotide is rapidly consumed, but incorporation into phospholipids is efficient because there is no competition with unlabelled nucleotide. In contrast, at high ATP concentrations, the label is incorporated at a slower rate (because of competition by unlabelled ATP), and no maximum level is reached during the incubation period. 4. PLC-like reactions slowly remove the radioactive phosphate from the lipids.

For the actin nucleation process it was important to analyze the changes in concentration of unlabelled lipids predicted by the model. With 200 μM ATP, PI and PIP were slowly consumed. The concentration of PIP2 was maintained at quite stable values (Fig. 4C, left panel). At higher ATP concentrations, this nucleotide and PIP were consumed at a slower rate, whereas the kinetics of PI consumption did not change significantly. PIP2 slightly increases during the incubation (Fig. 4C, right panel). DAG, the final product of reaction 3, 4, and 5, accumulated with similar kinetics at low and high concentrations of ATP (not shown).

### Modelling of the actin assembly process

Phagosomes are able to nucleate microfilaments *in vitro *when incubated in the presence of actin. During this process the actin monomers are somehow inserted into the filaments at the surface of the membrane and 'treadmill' outwards from this site towards the depolymerizing pointed end at the opposite side of the filament. In this poorly understood process, all the molecular machinery involved is present on the isolated phagosomes; no cytosolic components are necessary [7]. From previous experiments, it was known that lipids have a strong influence on this process. Fig. 2A shows the network of relevant lipids that were systematically tested in the actin nucleation assay. Other lipids not included in this network are also able to influence actin nucleation [8]; however, as a first approach, we only included lipids in a simple interconnected network whose time course of interconversion was experimentally accessible. Because of the well-established observation that phagosomes nucleate actin more efficient at low (0.2 mM) than at high (5 mM) concentrations of ATP, the whole set of lipids was tested at low and high concentrations of this nucleotide.

ATP and lipids probably affect actin nucleation through complex interactions with proteins. However, detailed molecular mechanisms for these interactions are still missing. We therefore adopted a phenomenological (black-box) approach to represent the empirical observations. As presently understood, actin filaments grow from membranes at specific nucleation sites. Therefore, we assumed that lipids and ATP would influence the number of active nucleation site on the membranes. Phagosomes were classified as positive (having one or more nucleation sites polymerizing actin) or negative (lacking any nucleation site polymerizing actin). Initial concentrations for lipids under control conditions (i.e., no lipid addition) were set as explained for sub-model II. From experiments with radioactive PC, PA, PIP, and PIP2, the amount of lipid incorporated in phagosomes after addition to the phagosomal fraction was estimated (see "Lipid incorporation into phagosomes" in Methods). PC has a strong inhibitory effect on actin nucleation at relatively low concentrations. Since this lipid is very abundant in the membranes, we reasoned that externally added PC and the endogenous PC may have different effects on actin nucleation and we therefore considered both as different species. Accordingly, PC in the control conditions was set to zero and to 0.8 μM after PC addition. We developed a minimal phenomenological model that was able to describe the observed experimental results.

$${\text{n}}_{\text{i}}\overset{{\text{v}}_{\text{a}}}{\underset{{\text{v}}_{\text{i}}}{⇄}}{\text{n}}_{\text{a}}\overset{}{\underset{{\text{v}}_{\text{g}}}{\to }}{\text{n}}_{\text{a}}\text{Actin}$$

This model assumes that actin nucleation sites can be in three configurations: inactive (unable to nucleate actin filaments, n_i_), active (able to nucleate actin filaments, n_a_) and polymerizing actin filaments (n_a_Actin). The nucleation sites are activated with a certain rate v_a _or inactivated with rate v_i_. Active nucleation sites (n_a_) initiate actin polymerization with rate v_g _proportional to the actual (time-variable) concentration. The nucleation sites that are growing actin filaments (n_a_Actin) cannot be inactivated. The amount of n_a_Actin on the preparation was estimated from the percentage of phagosomes growing actin filaments. Finally, from experimental observation, it was known that the number of positive phagosomes reaches a plateau after 15 min, and then remains stable for about one hour. Therefore, we looked for a model that leads to a plateau with this kinetics. The process was described by the following two-dimensional system of differential equations:

d(n_a_)/dt = v_a _- v_i _- v_g _(changes in the number of active nucleation sites with time)

d(n_i_)/dt = - v_a _+ v_i _(changes in the number of inactive nucleation sites with time)

with

v_a _= k_a _* f_i _* n_i _(rate of nucleation sites activation)

v_i _= k_i _* f_a _* n_a _(rate of nucleation sites inactivation)

v_g _= k_g _* n_a _(rate of actin filaments nucleation)

f_a _= exp {- (p_PI _* PI + p_PIP _* PIP + p_PIP2 _* PIP2 + p_PA _* PA*ATP) }

f_i _= exp {- (q_ATP _* ATP + q_PC _* PC + q_DAG _* DAG) }

The terms f_a _and f_i _stand for the modifying effect of ATP and lipids (if present at a certain concentration) on actin nucleation. The COPASI software allowed us to solve these equations on specified parameter and initial values, followed by least-square-guided parameter optimization (see Additional file 3). Several different formulations were tested for f_a _and f_i_. The negative exponential proved to be the most suitable. Least-square-optimization rendered "p" parameters for inhibitory factors (i.e., ATP, PC, and DAG) and "q" parameters for activating or neutral factors (i.e., PI, PIP, PIP2, and PA) very close to zero. Hence, these parameters were set to zero and the model simplified to a single parameter per factor ("p", for activating or neutral factors; "q" for inhibitory factors). With this simplified model, only positive parameters were obtained after optimization. For PI, a lipid with no effect on actin nucleation, "p" was also close to zero. f_a _and f_i _dynamically change with time as ATP is consumed and lipid concentrations change with time. Note that f_a _and f_i _have positive values limited to the 0-to-1 range. Hence, activators (PIP, PIP2, PA) affect actin nucleation by blocking v_i_, keeping the nucleation sites in the active form. In contrast, inhibitors (ATP, DAG, PC) slow down v_a_, preventing re-activation of these sites (Table 3). For PA, which has an activating effect only at high ATP concentrations, best fitting was observed using a single "p" parameter affecting an interaction product ATP*PA (Table 3).

**Table 3:** Parameter estimation for actin nucleation (sub-model III)

Parameter	units	Value	sensitivity (ΔlnSS/Δlnp)
pPI	l/μmol	0.00	-
pPIP	l/μmol	0.724	2.18
pPIP2	l/μmol	0.819	0.96
pPA*ATP	l^2^/μmol^2^	3.05E-05	1.08
qATP	l/μmol	0.00085	0.61
qDAG	l/μmol	0.312	3.28
qPC	l/μmol	1.83	0.36

The results of actin nucleation (mean of 3 to 5 independent experiments) in the presence of 0.2 or 5 mM ATP with the addition of different lipids and the COPASI program were used to estimate parameters. Sensitivity for each parameter was calculated as explained in Methods.

**Figure 5:**
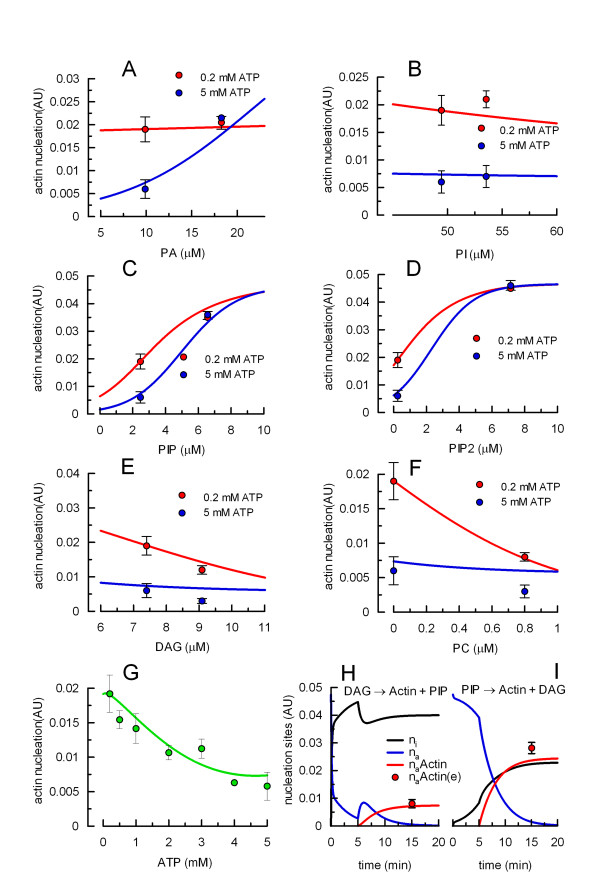
**Experimental and predicted values for actinnucleation**. A to F. Effect of ATP and lipids. Phagosomal preparations were incubated with actin and 0.2 mM (red) or 5 mM (blue) ATP. The percentage of phagosomes nucleating actin was measured under control conditions (estimated initial lipid concentrations in "Phagosomal lipid assumptions", Methods) or after addition of a single lipid (estimated initial concentration in "Lipid incorporation into phagosomes", Methods). For all panels, the percentage of positive phagosomes was used to estimate the number of active sites polymerizing actin (n_a_Actin) and expressed in arbitrary units (AU). G. n_a_Actin was estimated when the assay was supplemented with different concentrations of ATP. Symbols in panels A to G: experimental n_a_Actin ± SEM (N = 3–5); the model predictions are shown as solid lines having the same colours than the corresponding experimental observations. H. Phagosomal preparations were incubated first with DAG and 0.2 mM ATP. After 5 min, PIP was added to the assay together with actin, and the percentage of phagosomes nucleating actin was measured. I. Same protocol as in H, except that PIP was added during the preincubation and DAG together with actin. In H and I, the predicted number of inactive sites (n_i_, black lines), active sites (n_a_, blue lines) and active sites nucleating actin (n_a_Actin, red lines) are shown together with the experimental values [n_a_Actin(e), red circles ± SEM, N = 3].

Our model fitted most of the experimental data satisfactorily (Fig. 5A to 5F). Its deviation from the experimental data was about 13% (squared deviations) or 8% (absolute deviations), which indicates that the model has the flexibility to reproduce the experimental observation. One must keep in mind that the uncertainty in the lipid concentrations in the phagosomal membrane is large (see "Phagosomal lipid assumptions" and "Lipid incorporation into phagosomes" in Methods). However, by adjusting the "p" and "q" parameters, the model can correctly predict the changes in actin polymerization observed using a broad set of " initial" and "after addition" lipid concentrations (calculations not shown).

We have experimentally tested some non-trivial predictions of the model. It is important to stress that the set of parameters was not adjusted to fit the results of these experiments. The dependency of actin nucleation on ATP concentration was first assessed. As shown in Fig. 5G, the concentration-dependent inhibition curve predicted by the model closely followed the experimental data. We also tested an 'order-of-addition' experiment. The interesting feature in this protocol is that both conditions contain the same added reagents during the actin nucleation step. However, the model predicts poor actin nucleation when an inhibitory lipid is added first (in the absence of actin) and an activating lipid is added later together with actin. The inhibitory lipid prevents reactivation of inactive nucleation sites, which accumulate during the pre-incubation (v_a _< v_i_). When the activating lipid is added, it blocks v_i _and some sites are reactivated, but not enough to render an efficient actin nucleation process (Fig. 5H). In contrast, efficient nucleation is expected if the order of addition is reversed (i.e., first the activating lipid and then the inhibitory one together with actin). During the pre-incubation, the activating lipid prevents inactivation of active nucleation sites (v_a _> v_i_). When the inhibitory lipid is added it also blocks v_a_, but the active sites present are enough to polymerize actin filaments (Fig. 5I). Consistent with the model, low values of actin nucleation were observed when the inhibitory lipid was added first and high values when it was added later, together with actin (red circles in Fig. 5H and 5I). In conclusion, the simple model presented for actin nucleation on phagosomal membranes can account for all the lipid and ATP effects observed.

## Discussion

The membrane of the latex bead phagosomes isolated from J774 macrophages is a complex, metabolically active system. Here we initiated a global analysis of the dynamics of a sub-set of interactions that occur on phagosomes in response to ATP and different lipids that are involved in the regulation of *de novo *membrane-catalyzed actin assembly. Our results from a simplified bead-membrane phagosome system can be expected to provide the foundation for later investigations of the much more complex situation when the bead is replaced by a live pathogen such as *Mycobacterium tuberculosis*. A particularly challenging step in modelling the latex bead phagosome system was to select a qualitatively correct set of equations that accurately represent the processes occurring in the system. In a first step, we modelled a complex lipid network -containing most of the species we have identified that affect actin nucleation- interconnected by chemical reactions catalyzed by known enzymes (Fig. 2A). However, when experiments were performed, only the phosphatidylinositol branch of the network was found to be active. Several independent experimental lines of evidence support this conclusion, i) in the presence of radioactive ATP, ^32^P was incorporated almost exclusively in PIP and PIP2, ii) radiolabelled PA and PC were not actively metabolized by phagosomes, and iii) addition of unlabelled PI, PA, and DAG, only slightly affected the incorporation of ^32^P into lipids. These observations forced us to change the model to a more limited network of interconnected lipids (Fig. 2B). However, the problem was not completely solved. We could not find a set of parameters for the enzymatic reactions in the phosphoatidylinositol branch that reproduced the kinetics of incorporation of ^32^P into PIP and PIP2. A reaction in the model was missing and the data pointed to an ATP-consuming activity. When this reaction was included, the model reproduced the experimental data fairly accurately. The presence of an ATP-consuming reaction was tested experimentally; we clearly detected this activity and found that it was saturable, and that two independent reactions produced a better 'fit' of our experimental data. The latter were then used to parameterize what we refer to as sub-model I. The incorporation of^32^P into PIP and PIP2 by the phagosomal membrane reached a plateau after a few minutes. According to the model, the plateau was caused by depletion γ32P.ATP. Since the ATP hydrolytic activities were saturable, the model predicted that addition of unlabelled ATP should postpone the appearance of the plateau, a prediction that was confirmed experimentally. Among other data, we used in particular these experiments to adjust the parameter set for sub-model II.

In brief, the final set of equation described in sub-model I and II was established by a dynamic interplay between modelling and experiments. It is worthwhile noticing that the experiments were performed with several different phagosomal preparations over several months. Moreover, some experimental results reproduced observations published 6 years ago [4]. Therefore, we are confident that sub-model I and II faithfully represent the actual process of ATP consumption and lipid interconversion occurring in the phagosomal membrane.

A molecular mechanism connecting membrane lipids, ATP, and the actin nucleation process is still missing. We therefore adopted a phenomenological approach to represent the empirical observations. Three simple reactions were included in sub-model III. According to experimental data, actin filaments start growing from defined points in the membrane where G actin is recruited and assembled into an initial filament by a molecular complex that includes ezrin and/or moesin proteins in combination with N-Wasp (Marion et al, in preparation). Although the complexity of the actin assembly reactions on the membrane defies a simple model, it is reasonable to believe that these proteins are in equilibrium between an assembled, active complex, ready to start the actin nucleation process and inactive, partially assembled complexes. We propose that lipids and ATP influence the dynamic interconversion between active and inactive actin nucleation sites. The third reaction -which we propose to be irreversible- is the growing of a filament from an active nucleation site. Experimental observations indicate that the length of the actin filaments growing from the phagosomes increases with time, suggesting that once a nucleating site is polymerizing actin, it remains active for a relatively long period of time.

Several functions for the influence of lipids and ATP on actin nucleation were tested. The best fitting with a minimum number of parameters was obtained with a negative exponential function. With this simple equation, all the experimental data were satisfactorily fitted with a single positive parameter per lipid and ATP. Qualitatively, the exponential function can be approximately transformed and interpreted by setting exp{-p*P} = 1/(1+p*P) (p being an association constant and P the effector concentration), which is approximately valid if p*P is close to zero. This approximation displays lipids and ATP as inhibitors of the respective nucleation sites activation and inhibition reactions. The fact that the negative exponentials fit better than the inhibitor binding kinetics is probably due to inhomogeneous binding kinetics extending over a larger concentration range. The functional interaction between lipids, ATP, and nucleation site factors is complex, and the logarithmic concentration scale (implied by exponentiation) covered the phenomena better than any binding model based on linear concentration scales.

The uncertainty in the lipid composition on phagosomal membranes, both before and after lipid additions, is large; however, the model was reasonably robust to changes in these values. By adjusting the "p" and "q" parameters, reasonably good fitting was obtained assuming very different lipid concentrations. Analyzing the curve slopes in Fig. 5A to 5F, at physiological concentrations of ATP (about 5 mM) actin nucleation was very sensitive to changes in PA, PIP, and PIP2, suggesting that these lipids are necessary to prevent inactivation of active sites. In contrast, at reduced concentrations of ATP (i.e., 0.2 mM), the process was constitutively activated. Addition of PIP and PIP2 further stimulates actin nucleation while the inhibitory lipids DAG and PC strongly inhibit this process. It was surprising that PIP has a direct role in stabilizing actin nucleation sites and not an indirect effect through the generation of PIP2 that is in general the lipid that binds proteins participating in acting nucleation [15]. This latter lipid contributes to the negative charge of the lipid bilayer, a physical property that may be important to stabilize active nucleation sites [16]. PC is one of the most abundant lipids in biological membranes. However, external addition of PC to tissues or cell cultures has strong biological effects. For example, in Caco-2 cells, μM concentrations of PC in the medium have a remarkable anti-inflammatory effect [17]. Actin nucleation on phagosomes is also very sensitive to the addition of low concentrations of this lipid. The externally added lipid may interact with proteins in a different manner than endogenous PC [17] or the fatty acid composition of the added PC may be different. We have observed that PC carrying an arachidonic acid molecule (a strong activator of actin nucleation) is not inhibitory in the actin nucleation assay [17].

## Conclusion

Actin nucleation on phagosomes and its regulation by ATP and lipids involves numerous multi-component interactions. Our knowledge about the molecular and biochemical machinery active in this complex system is very limited. Moreover, our knowledge is not balanced: more is known about the biochemistry of the lipids interconversion than about the actin nucleation site dynamics. To overcome these difficulties, we have modelled the process using a three-step approach. Sub-models I and II are based on known chemical reactions connecting ATP and the lipid of interest. Experimental data were used to narrow the large set of possible reactions, allowing a satisfactory predictive model containing a few parameters. In contrast, for the third sub-model, a phenomenological description in terms of a very simple system of differential equations was developed. In this case also, a convenient fitting of the experimental data was obtained with a relatively small set of parameters. Although in its present form sub-model III works as a black box with no details about the factors involved, it can be easily decomposed to incorporate specific lipid or ATP influences on defined reactions. Experimental work assessing the role of specific proteins containing lipid-binding domains on actin polymerization will be necessary to unveil the molecular mechanism underlying the actin polymerization process modelled here.

## Methods

### Reagents

Phosphatidylinositol-4-phosphoate (from brain), Phosphatidylinositol-4,5-bisphosphate (from brain), diacylglycerol, and phosphatidylcholine were from Avanti Polar Lipids, Inc. (Alabaster, USA). Phosphatidic acid, and phosphatidylinositol were from Sigma Chemical Co. (St. Louis, USA). All other reagents were from Sigma Chemical Co. or Merck Chemicals (Darmstadt, Germany). Radioactive phosphatidylcholine (L-α-dipalmitoyl, [choline-methyl-^3^H]) and phosphatidic acid (L-α-dipalmitoyl, phosphatidic acid, - [glycerol-^14^C(U)]) were from PerkinElmer Inc (Waltham, USA).

### Actin nucleation assay by fluorescence microscopy

This assay has been previously described [7]. Briefly, glass slides were coated with 0.5% fish-skin gelatin in water and air-dried before the experiment. A constant number of phagosomes (Optical Density at 600 nm, OD_600 _= 10) was incubated between a slide and a coverslip in P buffer (20 mM HEPES, pH 7.0, 50 mM KCl, 4 mM MgCl_2_, 0.2 mM CaCl_2_, 0.2 mM ATP, 0.03% fish-skin gelatin, and protease inhibitors) with 2 μM rhodamine G-actin, 6 μM thymosin β4, and an antifade reagent at room temperature for 15 min. The proportion of positive phagosomes was established by counting at least three different microscope slides in a Zeiss Axioscope microscope (Zeiss, Oberkochen, Germany). The proportion was converted to number of nucleation sites polymerizing actin (n_a_Actin) considering that any positive phagosome has at least one n_a_Actin and that the distribution of nucleation sites per phagosome follows a Poisson distribution. We considered these numbers as arbitrary concentration units.

### Phagosomal lipid assumptions

Total protein concentration measured by Bradford's method in phagosomal preparations (OD_600 _= 10) was 1.2 ± 0.14 mg/ml (mean ± SD, N = 3). The phospholipid/protein ratio reported for synaptic vesicles is 0.515 (mg/mg) [18]. Assuming an average molecular weight for membrane lipids of 1000 Da [19], the molar concentration would be 0.515 μmol/mg of protein. A comparable concentration (0.282 μmol phospholipids/mg of protein) has been reported for latex bead-containing phagosomes from *Acanthamoeba castellanii *[20]. Hence, a 0.618 mM.phospholipid concentration was estimated for phagosomal preparations having 1.2 mg/ml of total proteins. Relative endogenous lipid molar ratios (referred to total phospholipid in the sample) in our phagosomal preparations were estimated from an ongoing lipidomic analysis of latex bead phagosomes: 43% PC, 8% PI, 1.6%PA, and 1.2%DAG (J. Brouwers and B. Helms, personal communication). Relative concentrations for PIP (0.4%) and PIP2 (0.04%) were estimated from the literature [21,22]. Endogenous lipid concentrations were then estimated as: 266 μM PC, 49 μM PI, 2.5 μM PIP, 0.25 μM PIP2, 10 μM PA, 7.4 μM DAG.

### Lipid incorporation into phagosomes

To estimate the efficiency of lipid incorporation, phagosomal preparation were incubated under the same conditions used in the actin polymerization assay with 50 μM PIP, 50 μM PIP2, 10 μM PA, or 1 μM PC containing 0.1 μCi of the corresponding radioactive lipid. After 5 min incubation at 20°C, the samples were centrifuged at 10.000 rpm for 5 min to pellet the phagosomes. The pellets were washed twice and the radioactivity was measured in pellets and supernatants. The results indicate that 8.2 ± 1.3% PIP, 13.8 ± 0.7% PIP2, 16.8 ± 0.3% PA, and 79.7 ± 0.9% PC were incorporated into phagosomes (mean ± range, N = 2 or 3). Incorporation of PI and DAG were assumed similar to those measured for PIP and PA, respectively. Therefore, considering that 1 μM PC, 10 μM DAG, 50 μM PI, 50 μM PIP, 50 μM PIP2, or 50 μM PA where used to modulate actin nucleation, the membrane concentrations after lipid addition were estimated as 54 μM for PI, 6.6 μM for PIP, 7.1 μM for PIP2, 18 μM for PA, and 9.1 μM for DAG. Since PC is abundant in the membranes and it affects actin nucleation at very low concentrations, we considered externally added PC (0.8 μM) and endogenous as different species.

### TLC separation of radioactive species

Forty μl aliquots of phagosomal samples were incubated as in the actin assembly assay with different concentrations of ATP and 40 μCi/ml of ^32^P γ-labelled ATP (10 mCi/ml, Amersham) at room temperature for different period of time. When indicated, lipids (labelled or unlabelled) or water soluble compounds were added. The reaction was stopped adding 100 μl 1 M HCl and 280 μl methanol/chloroform (1:1, vol/vol). The organic phase was collected, dried under vacuum and dissolved in chloroform/methanol/water (75:25:2, vol/vol). Subsequently, the lipids were separated by TLC on Silica Gel G60 plates [pre-treated with 1% potassium oxalate/2 mM EDTA in methanol/water (1:1, vol/vol)] using a solvent mixture of chloroform/acetone/methanol/glacial acetic acid/water (80:30:26:24:14, vol/vol) [23]. To measure ATP hydrolysis in the system, 1 μl of the water phase was loaded in the TLC plate. The position of phospholipid standards in the plate was determined as described [4]. The quantification of ^32^P-labelled phospholipids separated by TLC was performed with a Fujifilm Imaging Plate and Fujifilm Fluorescent Image Analyzer FLA-2000 equipment (Fujifilm, Elmsford, NY). As standard, a known amount of γ32P.ATP was spotted in the TLC plate.

### Network modelling

The mathematical model was formulated combining three sub-models: ATP decay, lipid phosphoryaltion/dephosphorylation, and actin nucleation. This was suggested by the fact that lipid phosphorylation amounted only to a very small fraction of ATP consumption and the actin nucleation did not feed back appreciably into the ATP and lipid metabolism.

Metabolites and chemical reactions were modelled in COPASI [24] assuming a single compartment. Internally, COPASI works with number of molecules. The concentration units we used can be transformed directly to molecule numbers by specifying the volume. However, the phagosomal preparation is a complex system where some interactions occur in a space almost restricted to two dimensions (the phagosomal membrane), other in the membrane-buffer interphase and other in the buffer. This complexity is not taken into account by COPASI. Notwithstanding, this heterogeneous system has some advantages. The lipid and protein concentrations on the phagosomal membrane are established by the cell and they do not depend on the efficiency of the purification procedure. These may explain the very consistent effect of lipids and ATP on actin nucleation observed in all phagosomal preparations tested. In sub-model III (actin polymerization), concentration units are arbitrary, a fact that does not disturb the calculations because we do not postulate stoichiometric relationships between lipid, ATP and actin nucleation sites.

Radioactively labelled and unlabelled metabolites were introduced as separate species sharing the same reactions (COPASI files are provided as Additional file 1, 2, and 3). Model parameters were estimated from comparison of the measured and predicted metabolite concentrations. The parameter set selected was the one that yielded a minimum of the sum of squared differences between experimental and predicted concentrations. As a routine, the "Particle Swarm", "Simulated Annealing", or "Nelder-Mead" methods provided by COPASI were used to obtain an initial set of parameters. The fitting was refined using the "Hooke and Jeeves" method with a tolerance of 10^-10^. With these method combinations, the same optimal parameters were obtained irrespective of the starting values indicating that local minima were avoided.

The quality of the fit for each sub-model was estimated by a dimensionless number expressing the deviation of the model from experimental data normalized by the mean fitted values. This allows comparison of different models and different data sets. The deviation of the model from experimental data was calculated from the sum of squared deviations between data and prediction (SS) provided by COPASI and from absolute deviations between fitted and experimental values. The square criterion is in keeping with the statistical theory of non-linear maximum-likelihood estimation. The absolute deviation criterion gives usually similar results and it is of more practical value since it is less affected by occasional large outlier values. The calculation formulas were: square root (SS/N)/(mean of fitted values) * 100 or (sum of ABS(experimental-fitted)/N)/(mean of fitted values) * 100. A low (< 20%) percentage value is indicative of a good fit.

COPASI provides, in addition to the best parameter set, standard deviation of the parameter estimates assuming a maximum-likelihood statistics, which in effect expands a quadratic surface of the sum-of-squares fit criterion around the optimal fit. This statistical model was not well-founded in our data (there was no symmetric ellipsoid around the minimum, and parameter estimates revealed interdependence of greatly varying degree). We therefore adopted a simple intuitive criterion of parameter certainty: we defined sensitivity for each parameter as the relative increase of the SS when the parameter was perturbed and hold constant (upward and downward with two increments, 1.5 and 2.25 folds). The rest of the parameters were "re-optimized" to minimize SS. To obtain a scale-free measure of this sensitivity, we calculated the quantity Δln SS/Δln "parameter perturbation". In Tables 1, 2, and 3, the average sensitivity when parameters were perturbed upward and downward by a factor of 2.25 is shown. A sensitivity value of 1.0 says that if a parameter is multiplied by a factor "n", the SS increases by the same factor. A small sensitivity (e.g. < 0.1) is indicative of loose determination of the parameter value by the experimental data. The sensitivity analysis indicates that the fits were satisfactory, but the estimates of some of the phenomenological parameter values were less certain.

COPASI can be downloaded from . The three sub-models, exported from COPASI as SBML files are included as "Additional Files".

## Authors' contributions

MK performed most of the actin nucleation experiments, contributed to the ^32^P incorporation studies, participated in the design of the study, and contributed to manuscript preparation. LSM performed most of the ^32^P incorporation experiments, participated in the design of the modelling strategy, carried out the models in COPASI, and drafted the manuscript. TD contributed to the design, conceived an early version of the actin nucleation model, and contributed to the manuscript preparation. JT contributed to the design and implementation of an early version of the actin nucleation model. RS contributed to the design and implementation of an early version of the actin nucleation model. EA performed part of the actin nucleation experiments. GG conceived the study and contributed to the manuscript preparation. JR designed the modelling strategy and contributed to the manuscript preparation. All authors read and approved the final version of the manuscript.

## Supplementary Material

Additional file 1 **Sub-model ATP consumption.** Model for ATP consumption in purified phagosomes Click here for file

Additional file 2 **Sub-model phospholipid metabolism.** Model for the active branch of the phospholipid metabolism in purified phagosomes Click here for file

Additional file 3 **Sub-model actin nucleation.** Model for lipid and ATP regulation of actin nucleation on isolated phagosomes Click here for file
